# Depression and Cardiac Disease: Epidemiology, Mechanisms, and Diagnosis

**DOI:** 10.1155/2013/695925

**Published:** 2013-04-07

**Authors:** Jeff C. Huffman, Christopher M. Celano, Scott R. Beach, Shweta R. Motiwala, James L. Januzzi

**Affiliations:** ^1^Harvard Medical School, Boston, MA 02114, USA; ^2^Department of Psychiatry, Massachusetts General Hospital, Blake 11, 55 Fruit Street, Boston, MA 02114, USA; ^3^Division of Cardiology, Department of Medicine, Massachusetts General Hospital, Boston, MA 02114, USA

## Abstract

In patients with cardiovascular disease (CVD), depression is common, persistent, and associated with worse health-related quality of life, recurrent cardiac events, and mortality. Both physiological and behavioral factors—including endothelial dysfunction, platelet abnormalities, inflammation, autonomic nervous system dysfunction, and reduced engagement in health-promoting activities—may link depression with adverse cardiac outcomes. Because of the potential impact of depression on quality of life and cardiac outcomes, the American Heart Association has recommended routine depression screening of all cardiac patients with the 2- and 9-item Patient Health Questionnaires. However, despite the availability of these easy-to-use screening tools and effective treatments, depression is underrecognized and undertreated in patients with CVD. In this paper, we review the literature on epidemiology, phenomenology, comorbid conditions, and risk factors for depression in cardiac disease. We outline the associations between depression and cardiac outcomes, as well as the mechanisms that may mediate these links. Finally, we discuss the evidence for and against routine depression screening in patients with CVD and make specific recommendations for when and how to assess for depression in this high-risk population.

## 1. Introduction

Depression in cardiac disease is common, persistent, underrecognized, and deadly. Over the past 20 years, research has found that not only is depression more common in cardiac patients than in the general population, but depression is also a risk factor for cardiac morbidity and mortality, independent of traditional risk factors. This link between depression and cardiac morbidity likely involves both physiologic and behavioral effects of depression. Fortunately, screening for depression in cardiac patients is straightforward, and methods of diagnosis essentially parallel those for major depressive disorder (MDD) diagnosis in other settings. In this paper, we will review the epidemiology, course, impact, pathogenesis, and diagnostic assessment of depression in patients with cardiovascular disease (CVD). We will define *depression* as either elevated depressive symptoms on a validated depression scale or a formal diagnosis of MDD. Of note, we will not specifically address treatment of depression in cardiac patients, a topic that is worthy of its own separate review and that has been recently reviewed in detail [1].

## 2. Prevalence of Depression in Cardiac Patients

Depression is highly prevalent in cardiac patients. Between 31–45% of patients with coronary artery disease (CAD), including those with stable CAD, unstable angina, or myocardial infarction (MI), suffer from clinically significant depressive symptoms [2]. Furthermore, 15–20% of patients with CAD meet criteria at any given time for the full syndrome of MDD [3–7]; this rate of MDD is roughly threefold higher than in the general population [8] and is similar to the rates of MDD in patients with chronic kidney disease [9] and cancer [10].

Patients with heart failure (HF), atrial fibrillation (AF) and those undergoing implantable cardioverter-defibrillator (ICD) placement are similarly at increased risk for elevated depressive symptoms and for MDD [11–13]. A meta-analysis of patients with HF found prevalence rates of 36% for increased depressive symptoms and 20% for MDD [12]. Furthermore, a recent systematic review of ICD patients found depressive disorders (e.g., MDD, dysthymic disorder) to be present in 11–28% of patients [11]. Finally, among patients undergoing coronary artery bypass graft (CABG) surgery, approximately 30–40% of patients meet criteria for dysthymia, minor or major depression, with roughly 15% of patients meeting full MDD criteria on diagnostic interview [14]. 

## 3. Course of Illness

In patients with CVD, depression is often chronic and recurrent. Among patients with CVD hospitalized for acute cardiac events and found to meet criteria for depression during or shortly after admission, approximately 50–70% had ongoing depressive symptoms that preceded their cardiac event [15–17]; this finding is consistent with literature that describes persistent depression in patients with stable CAD [18]. Furthermore, rather than being a transient reaction to a cardiac event, depression for many patients exists for months or years before and persists long after the event [15–22]. In studies that examine the course of post-MI depression, depressive symptoms remain at steady levels of severity over the 12 months after an MI [19, 20]. Similar results have been observed in patients with chronic CVD, such as those with ICDs; in this cohort, 80% of patients who are depressed at the time of ICD placement continue to suffer from depressive symptoms 2 years later [22]. Finally, among patients admitted with a HF exacerbation and diagnosed with MDD, less than half have a remission of symptoms at 5-month followup [21]. 

In sum, depression is present in a significant portion of patients across the spectrum of cardiac disease, and such symptoms, when present, are likely to persist unless treated. These findings underscore the need to find better methods for identifying—and managing—depression in patients with CVD. 

## 4. Risk Factors for Depression in Cardiac Patients

Though there are some inconsistencies in the literature, there are several established risk factors for depression in cardiac patients. Most studies have found that younger patients, women, and patients with premorbid histories of depression are more likely to have depression in the context of CVD [25–29]. Among patients suffering from an acute coronary syndrome (ACS), in addition to the previous factors, social isolation, prior ACS, and in some cases, comorbid diabetes may also increase depression risk [27, 28, 30–32]. 

These factors, as well as poor functional status or worse New York Heart Association (NYHA) HF severity class, have also been linked to depression in patients with HF [33–35]. Among CABG patients, depression pre-CABG is predicted by female gender, younger age, living alone, and less education [36], and post-CABG depression is best predicted, not surprisingly, by pre-CABG depressive symptoms and anxiety [37]. Similarly, among patients with an ICD for ventricular arrhythmias, younger age and female gender predict depression [11]; there is some suggestion, though not definitive evidence, that ICD shocks are linked to higher rates of depression [11, 38]. 

## 5. Psychiatric Conditions Commonly Comorbid with Depression in Cardiac Patients

With respect to comorbid symptoms and conditions, symptoms of anxiety and formal anxiety disorders are strongly correlated with depression in a wide variety of cardiac populations, as they are in the general population. Elevated symptoms of anxiety are highly prevalent in cardiac patients, including those suffering an ACS [39, 40], those with stable CAD [41], those participating in cardiac rehabilitation [42], and those hospitalized for one of several different cardiac conditions [17]. 

Formal anxiety disorders are frequently seen in a range of cardiac patients as well [43]; of these disorders, generalized anxiety disorder (GAD) is the most common by far. For example, GAD was concomitantly present in 38% of MDD cases in a large study of outpatients with CAD [44], and GAD was commonly comorbid with MDD among patients suffering an ACS [45].

In addition to GAD, posttraumatic stress disorder (PTSD) is common among specific populations of depressed cardiac patients, especially CABG patients and those receiving ICDs. Among patients undergoing CABG, PTSD occurs in approximately 15% of patients [14, 46], and a recent trial found that over half of post-CABG patients with PTSD had concomitant depression [46]. PTSD is at least common (18–37% in two identified studies) among patients with an ICD and is commonly comorbid with depression [47, 48]. In these patients, PTSD may stem from the cardiac event necessitating ICD placement or from firing of the device itself [49].

Anxiety disorders, especially GAD, may be associated with mortality and other adverse cardiac outcomes, independent of both traditional risk factors and depression [41, 44, 50, 51], and thus these disorders that are so often comorbid with depression should also be addressed. There is controversy regarding whether the effects of GAD and MDD on cardiac outcome are additive, with some [50], but not all [41], studies finding additive effects. Assessment for anxiety symptoms can be completed via the Hospital Anxiety and Depression Scale (which has the advantage of also containing a depression subscale), while specific screening for GAD can be completed using the Generalized Anxiety Disorder-2 [52] (GAD-2) or Generalized Anxiety Disorder-7 [53] (GAD-7) measures.

## 6. Association between Depression and Cardiac Outcomes 

A multitude of studies over the past 15 years have confirmed that depression is associated with adverse cardiovascular outcomes, independent of traditional risk factors. In healthy individuals, depression has been independently associated with the development and progression of CAD [54–57] and with CVD-related mortality [58]. In fact, two separate systematic quantitative reviews have found depression (diagnosed with a diagnostic interview or self-report measure) to be a significant and independent risk factor for the development of cardiac disease, with a relative risk of 1.6 (relative risk 1.64 [95% Confidence Interval (CI) = 1.41–1.90] and 1.60 [95% CI 1.34–1.92], resp.) in depressed patients compared to those persons who were never depressed [56, 59]. 

Depressed patients with unstable CAD appear to be at even greater risk for poor cardiac outcomes. The presence of post-MI depression predicts recurrent cardiac events [60], cardiac-related death [60–63], and all-cause mortality [60, 64]. Indeed, a recent meta-analysis revealed that depressed post-MI patients have a 2.4-fold increased risk (unadjusted) for all-cause mortality (odds ratio 2.38; 95% CI = 1.76–3.22) [60]. Likewise, among patients with a wider range of unstable or angiographically validated CAD, a meta-analysis of 20 studies found depressive symptoms and MDD to be associated with mortality (Hazard ratio 1.76 [95% CI = 1.27–2.43], adjusted for other risk factors) in the two years following an event [64]. Furthermore, depressive symptoms following MI have been associated with increased hospital readmissions, particularly cardiac readmissions, and with reduced adoption of secondary prevention behaviors, including smoking cessation, physical activity, and cardiac rehabilitation [65–68].

Regarding the timing of depression onset, a 2012 meta-analysis found that depression was independently predictive of mortality and cardiac morbidity after an ACS, regardless of whether the depression was present prior to or after the onset of cardiac illness; however, first-onset depression within 30 days of an acute cardiac event was potentially more strongly linked to morbidity and mortality [69]. Of note, following an acute cardiac event, prior history of depression, without current depression, was not associated with adverse outcomes [69].

Depression also appears to substantially impact cardiovascular outcomes in patients with other forms of CVD. Clinically significant depression increases the risk of incident HF, especially in those already at increased risk for its development; in patients with established HF, it is also related to increased health care utilization, more frequent hospitalizations, and a 2-fold increase in mortality risk [12, 70]. Increased depressive symptoms are also linked to recurrence of AF in patients following cardioversion [71] and with cardiovascular mortality in patients with comorbid AF and HF [72].

In patients undergoing CABG, depression has been associated with longer hospitalization [73], poorer functional outcomes [74, 75], more perioperative complications [73], worse health-related quality of life (HRQoL) [76], progression of atherosclerotic disease [77], higher rates of rehospitalization [75], and mortality [14, 78, 79]. Finally, depression has been associated with mortality, independent of covariates, in patients with an ICD [80]. 

Several studies have evaluated the characteristics of psychiatric illness and its treatment that may contribute to poor cardiac outcomes in depressed cardiac patients. Nonresponse to treatment for depression, for instance, appears to put depressed post-ACS patients at greater risk for recurrent cardiac events [81] and for all-cause mortality [82]. Poor outcomes may be exacerbated by the presence of cooccurring anxiety, which is independently associated with recurrent cardiac events and mortality [83] and which has been linked with poor response to treatment for depression [84–86]. Other patients who appear to be at particularly high risk for poor outcomes include those with prominent anhedonia (the inability to experience pleasure) [87–89] and those with type D personality (a personality structure characterized by negative affectivity and social inhibition) [19], though the latter association is controversial [90].

Thus, not only is depression common and persistent in patients with CVD, but it also may have a negative impact on multiple aspects of the course of cardiovascular illness, including physical functioning, quality of life, health care utilization, rehospitalization, and mortality. 

## 7. Potential Mechanisms Linking Depression and Cardiac Disease (Figure 1)

 If indeed depression is an independent predictor of cardiac illness, it is important to understand the physiologic and behavioral underpinnings of this association. Indeed, there are a number of mechanisms that are potentially implicated in the connection between depression and adverse cardiac outcomes. 

### 7.1. Inflammation

 The contribution of inflammation to the overall development of cardiac disease—and especially to acute cardiac events—is well documented. Inflammatory cytokines have been associated with atherosclerotic plaque formation, progression, and rupture; as such they are major contributors to the pathogenesis of CAD, unstable angina, and MI [91, 92]. Furthermore, inflammation plays a key role in the pathogenesis of certain types of HF [93, 94]. Overall, inflammatory cytokines (i.e., C-reactive protein (CRP) in CAD and interleukin-6 (IL-6) in HF) have been predictive of cardiovascular mortality and disease progression in healthy individuals [58] and in patients with CAD [95] and HF [96–98].

Depression also has been linked to increased levels of cytokines (specifically CRP, IL-1, and IL-6), both in patients with and without a history of cardiac disease [54, 99–101]. Two studies provide evidence that inflammation associated with elevated depressive symptoms or MDD is associated with the development of cardiac disease and cardiovascular mortality. In a population cohort study of 908 patients without known CVD, Kop and associates [58] found that depression predicted cardiovascular mortality; controlling for inflammatory markers reduced the association by 12.7%, suggesting that inflammation partially contributed to the effects of depression on cardiovascular mortality. Similarly, in a study of 559 women with suspected cardiac ischemia, Vaccarino and associates found that depression predicted cardiovascular events; controlling for inflammatory factors (CRP, IL-6) reduced this association by 20%, again suggesting a small but meaningful contribution to the effects of depression on cardiac events [102].

There are at least two potential mechanisms by which inflammation, depression, and cardiovascular disease may be linked. First, neural-immune interaction may occur. In animal models of induced fatigue, levels of the inflammatory cytokine interferon alpha increase, while extracellular levels of serotonin increase in the medial prefrontal cortex. Furthermore, treatment with a serotonin (5HT-1A) receptor agonist reduces the effects of fatigue [103, 104]. Hence, in depression, reduced serotonin actions on these receptors may be linked to increased cytokines and the subsequent effects on cardiovascular outcome. Second, elevated levels of inflammatory cytokines (e.g., interferon-gamma) are associated with increased activity of an enzyme that degrades tryptophan (a serotonin precursor) to kynurenine in patients with CVD [105]. This is likely to result in lower levels of serotonin and may represent another mechanistic link that connects inflammation to depression in patients with cardiac disease.

### 7.2. Endothelial Dysfunction

Endothelial dysfunction has been linked to the development of ischemic CAD in patients with atherosclerosis [106]. For example, while a normal endothelium typically releases nitric oxide in response to serotonin to ensure adequate blood flow through the coronary arteries, in atherosclerotic arteries it fails to do so. This results in vasoconstriction in areas of atherosclerosis and may provide a mechanism for myocardial ischemia and coronary thrombosis [107]. Inflammation, which has been associated with CAD, also impairs endothelial nitric oxide release and may represent a mechanism explaining the finding of endothelial dysfunction in cardiac patients [108]. In addition to its role in cardiac ischemia in patients with CAD, endothelial dysfunction independently predicts mortality in patients with HF [109, 110].

Depression also has been associated with impaired endothelial function in healthy patients [111, 112], in those at risk for CVD [113], and in those with established CVD [114]. Treatment of depression with selective serotonin reuptake inhibitors (SSRIs) has led to improved endothelial function in patients with depression and established CAD, further suggesting that endothelial dysfunction may be linked to depression's effects on cardiac outcomes [115].

### 7.3. Increased Platelet Activity and Aggregation

 Platelet adhesion, activation, and aggregation are important components of cardiac disease, and increased platelet activity may lead to coronary events on this basis. Serotonin plays a key role in platelet biology through its binding with 5-hydroxytryptamine (5-HT) receptors on platelets. In atherosclerotic arteries, as described in the previous section, serotonin leads to platelet aggregation [106, 107]. Furthermore, elevated levels of blood serotonin predict CAD and future ischemic cardiac events in patients with suspected CAD [116]. SSRIs, which theoretically deplete platelet serotonin stores by inhibiting platelet uptake of serotonin, have also been shown to decrease platelet aggregation and activity in vitro and in patients with CAD [117, 118]. Taken together, these findings lend credence to the theory that serotonin, through its activity on platelet aggregation, is associated with myocardial ischemia and other cardiac events.

 Platelet dysfunction also occurs in patients suffering from major or minor depression; depressed patients have abnormalities in whole blood and platelet serotonin levels [119], increased platelet serotonin receptor concentrations [120, 121], and abnormally low platelet serotonin transporter levels [122], suggesting that their platelets are both more sensitive to serotonin and less able to remove it from the bloodstream. Furthermore, there is evidence—albeit mixed—suggesting that the platelets of depressed patients are hyperactive [119, 123–125]. This serotonergic and platelet dysfunction could mediate the increased risk for ischemic events in these patients. At this stage, much less is known about the association between platelet hyperaggregability and other forms of CVD.

### 7.4. Neurohormonal and Autonomic Nervous System Dysfunction

 Neurohormonal activation may play a particularly important role in the connection between depression and outcomes in HF. Levels of circulating catecholamines (e.g., epinephrine and norepinephrine) are elevated in patients with HF, especially in those with decompensated HF, and higher levels of norepinephrine have been linked to greater mortality in this illness [126, 127]. Furthermore, increases in plasma as well as cerebrospinal fluid levels of norepinephrine have been observed in patients with MDD to the extent of being capable of causing increased mortality in HF [128]. Abnormalities in the hypothalamic-pituitary-adrenal (HPA) axis may also play a substantial role, as cortisol (and aldosterone) are independently linked with mortality in HF, and patients with depression have elevated levels of cortisol [98]. Such hypercortisolemia and other HPA-related abnormalities in depression may impact medical outcomes in other cardiac illnesses, as these abnormalities are associated with the development and progression of the metabolic syndrome, a condition characterized by dyslipidemia, truncal obesity, and insulin resistance and linked to cardiac morbidity and mortality. 

Beyond elevated levels of circulating catecholamines and cortisol, other abnormalities in the autonomic nervous system may also contribute to the relationship between depression and cardiac disease. Since the heart is innervated by both the sympathetic and parasympathetic nervous systems, the interplay between these two opposing forces helps the heart make changes in response to stressors. Patients with a history of ischemic heart disease or HF typically exhibit a pattern of increased sympathetic and decreased parasympathetic activity; this is manifested by decreased baroreflex sensitivity and decreased heart rate variability (HRV) [129]. This pattern of autonomic dysfunction has been associated with increased mortality in patients with HF [129] and a history of MI [129–131] and with increased rates of recurrent AF after cardioversion [71]. In animal studies, such a pattern of autonomic dysfunction was associated with increased rates of ventricular fibrillation during recurrent ischemic episodes [132] and may represent a mechanism by which autonomic dysfunction leads to increased morbidity and mortality in cardiac patients.

Depressed patients (with and without cardiac disease) also have reduced HRV [133–135], suggestive of the same imbalance between sympathetic and parasympathetic nervous systems described previously. This reduction appears to be linearly associated with depression severity, with more severe depression resulting in greater reductions in HRV [133]. Furthermore, patients with both CAD and depression have greater decreases in HRV compared to patients with depression or CAD alone, suggesting that the effects of depression and CAD on HRV are additive [135]. This increased autonomic dysfunction in depressed patients may therefore lead to worse cardiac outcomes in patients with cooccurring HF. 

### 7.5. Effects of Brain-Derived Neurotrophic Factor (BDNF) and Related Factors

In addition to the previous mechanisms, BDNF may also play an important role in the connection between depression and cardiac outcomes. Depression has been strongly and consistently linked to low levels of BDNF [136], and it is thought that BDNF signaling mediates the hippocampal neurogenesis that has been linked to depression recovery [137]. Indeed, SSRI antidepressants have been associated with increased levels of BDNF [138] and with hippocampal neurogenesis [139]. BDNF also has an important role in several physiologic processes important to cardiovascular health. BDNF is expressed by endothelial cells, and it leads to angiogenesis in, and survival of, endothelial cells (primarily mediated via the phosphatidylinositol-3-kinase-Akt pathway), with increased BDNF expression during hypoxia [140]. Endothelial cells are vital to vascular health and, as noted, endothelial function is independently associated with cardiac outcomes. 

Furthermore, BDNF expression is upregulated by neural signals from the heart after experimentally induced MI (interestingly, BDNF expression is increased in brain but not heart), and such expression was linked to reduced cardiomyocyte death and improved systolic function [141]. Such heart-brain connections are similarly seen with the brain sigma-1 receptor (S1R) [142]. Brain S1R appears to be associated with depression, as S1R knockout mice display depressive phenotypes [143], and S1R agonists improve such behavior [144]. A recent mouse model study showed that induced heart failure was associated with reduction in brain S1R, consistent with the investigators' hypothesis that reduced brain S1R exacerbates heart failure [145].

Finally, BDNF may be an important mediator of the previously noted HPA axis effects on depression and cardiovascular disease. The glucocorticoid receptor interacts with the specific receptor of BDNF, TrkB, and excessive glucocorticoid interferes with BDNF signaling [146]; therefore excess glococorticoids may be associated with adverse outcomes via BDNF-mediated effects on endothelial cells and cardiomyocytes.

### 7.6. Behavioral Factors

 Behavioral factors are undoubtedly involved in the relationship between depression and cardiac disease. Depressed patients are less likely to engage in health-promoting behaviors, including maintenance of a healthy diet [66, 147], regular exercise [66, 148], adherence to medications [66, 147, 149], stress reduction [66], and completion of cardiac rehabilitation programs [150, 151] following MI. These patients also have more difficulty lowering their cholesterol following MI [152]. Medication nonadherence and lower physical fitness are associated with an increased risk of cardiovascular events in certain populations [153, 154], and this additionally suggests that the behavioral changes associated with depression may be associated with the progression of CAD and poor cardiac outcomes in patients with and without established CVD. Fortunately, in a study of hospitalized patients with a variety of cardiac conditions, those who met criteria for clinical depression during admission had improvement of adherence (to diet, exercise, and medication) if their depression improved following hospitalization [147]. This suggests that reduced adherence to key secondary prevention behaviors in depressed cardiac patients may be modifiable with treatment of the depressive symptoms.

## 8. Identification of Depression in Cardiac Patients

Despite the existence of effective and safe treatments for depression in cardiac patients, depression remains underrecognized and undertreated in this population [155, 156]. In one study of post-MI patients, less than 15% of depressed patients were accurately identified as such by their treatment teams, and only 11% received treatment with antidepressants [155]. Given the increased morbidity and mortality associated with depression, it is important that these patients be more consistently identified.

Routine screening of cardiac patients for depression is one potential way to improve detection of depression in this patient population. The American Heart Association recommends such screening using the 2- and 9-item Patient Health Questionnaires (PHQ-2 and PHQ-9, resp.) [23, 24, 157], two brief screening tools for depression (see Tables 1 and 2). The PHQ-2 inquires about the presence and frequency of depressed mood and anhedonia, while the PHQ-9 includes questions about the nine diagnostic criteria for MDD (the first two questions of the PHQ-9 are the PHQ-2). Both screening tools are time-efficient and have the potential to be integrated into standard inpatient and outpatient evaluations. Furthermore, elevated scores on these scales have prognostic value; for example, positive screens using this two-step method have been independently associated with subsequent cardiac events in outpatients with CAD [158], and a positive PHQ-2 screen has been associated with subsequent mortality among patients with HF [159]. 

Regarding the operating characteristics of these tools in cardiac patients, a large study of outpatients with CAD found that the PHQ-2 (with a cutoff score of 2) had sensitivity of 82%, specificity of 79%, positive predictive value of 52%, and negative predictive value of 94% (using a contemporaneous diagnostic interview for MDD as the gold standard), making it a reasonably good screening tool in this cohort, especially given its substantial brevity and excellent ability to exclude MDD [160]. In the same cohort, PHQ-9 (using the standard cutoff score of 10) had sensitivity of 54%, specificity of 90%, positive predictive value of 61%, and negative predictive value of 88% [160]. The recommended two-step screening approach using both items did not appear to be superior to using either tool alone for screening [160]. However, a related analysis from the same study found that use of the two-step screening with PHQ-2 followed by PHQ-9 enabled nearly half of the sample to avoid undergoing the full PHQ-9 scale with few patients with “true” depression missed as a result [158]. 

Clinical implementation of routine, universal screening with the PHQ-2 or PHQ-9 has had some success but overall mixed results. One study of this two-step screening method found relatively high rates of completion and high staff satisfaction among patients hospitalized on inpatient cardiac units. However, in this case, the PHQ-2 screening was performed by clinical nurses as part of routine care, while the follow-up PHQ-9 was performed by a study social worker [161]. A more complete clinical implementation of the two-step screening resulted in substantial detection of depression in screened patients but only modestly improved overall recognition of depression due to a significant minority of patients who did not get screened; clinical staff reported preference for a single-stage screen instead [162]. A different approach to screening—once-yearly screening with the PHQ-9—at a large veterans affairs medical center—was associated with low rates of depression detection in cardiac patients compared to prior studies, and reliance on such archival screening may miss patients with newer-onset depression in the setting of cardiac events [25]. 

However, the question of whether to screen at all is a vital one. There has been much controversy about the AHA recommendations, in large part because there is no evidence that routine screening alone for depression, whether in the general population or in cardiac patients, improves patient outcomes. Critics of universal screening have, therefore, suggested that the recommendations of the AHA be reconsidered [6, 163]. Specific concerns have included potential misdiagnosis of patients with screening tools if patients with positive screens do not undergo confirmatory psychiatric interviews, unnecessary stigma for patients who may be misdiagnosed, and lack of evidence regarding the cost effectiveness of screening programs [163].

In contrast, when depression screening is paired with a management protocol or system of care (e.g., a care management program) to treat depression in persons with cardiovascular disease, there has been consistent evidence for improved patient outcomes (including, in some studies, improved adherence, reduced cardiac symptoms, reduced cardiac events and improved blood pressure and lipids) [164–167]. Thus, some proponents of universal screening suggest that systematic screening should be performed when there is an available and consistent resource for treatment available for patients who screen positive, such as collaborative care programs [168]. Such a policy would ensure that positive-screen patients would receive a more thorough depression evaluation and, if depression were diagnosed, would be able to obtain longitudinal treatment.

## 9. Clinical Recommendations Regarding Assessment

*Screening.* Where does this leave clinicians in the assessment of depression in cardiac patients? First, we agree with the assertion that depression screening should only be performed if there is a clear path to the patient getting treatment. This may take several forms: a systematic care management program, a trusted psychiatric colleague who can reliably follow the patient, or treatment directly from the primary medical team if they feel comfortable managing depression. Without the existence of such treatment options, systematic depression screening requires valuable resources (e.g., physician and nurse time) without clear benefit and may identify a new and important condition without offering adequate treatment. 

When performing screening, as noted, one-time screening with the PHQ-9 appears ineffective and inefficient, while two-stage screening with the PHQ-2 followed by the PHQ-9 appears to allow for most nondepressed patients to end the screening process after only two questions while using all nine DSM-IV criteria to assess positive PHQ-2 patients. In our opinion, an approach that uses single-step screening with the PHQ-9 but halts screening with negative responses to the first two items—the items that compose the PHQ-2—may find the best balance. 

*Diagnosis*. In patients who screen positive, how does one make a diagnosis of depression? Significant controversy exists regarding the diagnosis of depression in cardiac patients. This is especially true in patients who have had acute cardiac events. These patients may have significant anxiety and transiently depressed mood immediately following the event but may not develop MDD. Furthermore, symptoms related to the cardiac event (e.g., fatigue, poor concentration, poor appetite) may overlap significantly with the clinical symptoms of depression. As a result, some have argued for lengthening the amount of time needed for a depressive episode to be identified in patients with acute cardiac events, or for excluding somatic symptoms that could be attributed to cardiac disease. 

Despite these concerns, we would recommend continuing to use the DSM-IV criteria (including the 2-week duration criterion) for the diagnosis of depression, even in patients who have had an acute cardiac event. While one could argue for waiting longer than 2 weeks in post-ACS or post-CABG patients, the toxic effect of depression on cardiac health is relatively clear, and there is some suggestion from a recent meta-analysis that depression within 30 days of ACS (especially if it is new-onset depression) is more strongly associated with mortality and cardiac morbidity than premorbid depression or depression diagnosed over the first year [69]. 

Furthermore, using the DSM-IV criteria for MDD requires either loss of pleasure or depressed mood most of the day, nearly every day, for at least 2 weeks, and patients who meet this criterion, in addition to meeting other criteria (which may include somatic symptoms) in our mind, are very likely to have MDD, even in the face of cardiac illness. 

While beyond the scope of this review, safe and effective medication (e.g., sertraline, which is the agent most studied in patients with acute cardiac illness [169, 170]) and psychotherapeutic (e.g., cognitive behavioral therapy, successfully used in patients with substantial cardiac illness [171, 172]) treatments are available for cardiac patients with MDD. Therefore, patients who meet full criteria for MDD should be treated, whether cardiac events are recent or remote. We hope that systematic screening and treatment of depression in patients with CVD can lead to improved psychiatric and medical outcomes in this high-risk population. 

## Figures and Tables

**Figure 1:**
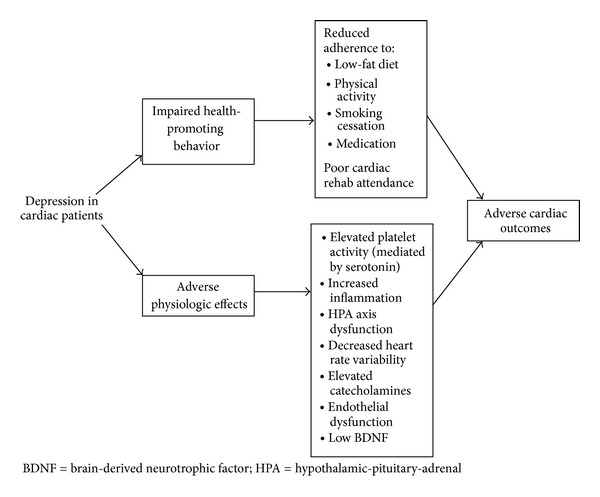
Mechanisms by which depression may impact cardiac outcomes.

**Table 1:** Patient Health Questionnaire 2 (PHQ-2) [23]*.

Over the past two weeks, how often have you:				
	Not at all	Several days	More than half the days	Nearly every day

Lost interest or had little pleasure in doing things	0	1	2	3
Felt down, depressed, or hopeless	0	1	2	3

Total score = sum of two items.

PHQ-2 score ≥ 3 is suggestive of elevated symptoms of depression.

*The PHQ-2 was developed by Drs. Robert L. Spitzer, Janet B.W. Williams, Kurt Kroenke, and colleagues, with an educational grant from Pfizer Inc. *PHQ2 Copyright *©* Pfizer Inc. All rights are reserved.*

**Table 2:** Patient Health Questionnaire 9 (PHQ-9) [24]^†^.

Over the past two weeks, how often have you been bothered by the following?					

		Not at all	Several days	More than half the days	Nearly every day

Little interest or pleasure in doing things		0	1	2	3
Feeling down, depressed, or hopeless		0	1	2	3
Trouble falling or staying asleep or sleeping too much		0	1	2	3
Feeling tired or having little energy		0	1	2	3
Poor appetite or overeating		0	1	2	3
Feeling bad about yourself or that you are a failure or have let yourself or your family down		0	1	2	3
Trouble concentrating on things, such as reading the newspaper or watching television		0	1	2	3
Moving or speaking so slowly that other people could have noticed. Or the opposite—being so fidgety or restless that you have been moving around a lot more than usual		0	1	2	3
Thoughts that you would be better off dead or of hurting yourself in some way.		0	1	2	3

If you checked off any problems, how difficult have these problems made it for you to do your work, take care of things at home, or get along with other people?					

Not difficult at all □	Somewhat difficult □	Very difficult □		Extremely difficult □	

Total score = sum of 9 items.

^†^The PHQ-9 was developed by Drs. Robert L. Spitzer, Janet B.W. Williams, Kurt Kroenke, and colleagues, with an educational grant from Pfizer Inc. *PHQ9 Copyright*  ©  *Pfizer Inc. All rights are reserved.*

## References

[1] Mavrides N, Nemeroff C (2013). Treatment of depression in cardiovascular disease. *Depression and Anxiety*.

[2] Celano CM, Huffman JC (2011). Depression and cardiac disease: a review. *Cardiology in Review*.

[3] Carney RM, Freedland KE (2008). Depression in patients with coronary heart disease. *The American Journal of Medicine*.

[4] Lespérance F, Frasure-Smith N, Juneau M, Théroux P (2000). Depression and 1-year prognosis in unstable angina. *Archives of Internal Medicine*.

[5] Schleifer SJ, Macari-Hinson MM, Coyle DA (1989). The nature and course of depression following myocardial infarction. *Archives of Internal Medicine*.

[6] Thombs BD, de Jonge P, Coyne JC (2008). Depression screening and patient outcomes in cardiovascular care: a systematic review. *The Journal of the American Medical Association*.

[7] Thombs BD, Bass EB, Ford DE (2006). Prevalence of depression in survivors of acute myocardial infarction: review of the evidence. *Journal of General Internal Medicine*.

[8] Kessler RC, Berglund P, Demler O (2003). The epidemiology of major depressive disorder: results from the national comorbidity survey replication (NCS-R). *The Journal of the American Medical Association*.

[9] Hedayati SS, Bosworth HB, Kuchibhatla M, Kimmel PL, Szczech LA (2006). The predictive value of self-report scales compared with physician diagnosis of depression in hemodialysis patients. *Kidney International*.

[10] Snyderman D, Wynn D (2009). Depression in cancer patients. *Primary Care—Clinics in Office Practice*.

[11] Magyar-Russell G, Thombs BD, Cai JX (2011). The prevalence of anxiety and depression in adults with implantable cardioverter defibrillators: a systematic review. *Journal of Psychosomatic Research*.

[12] Rutledge T, Reis VA, Linke SE, Greenberg BH, Mills PJ (2006). Depression in heart failure. A meta-analytic review of prevalence, intervention effects, and associations with clinical outcomes. *Journal of the American College of Cardiology*.

[13] McCabe PJ (2010). Psychological distress in patients diagnosed with atrial fibrillation: the state of the science. *Journal of Cardiovascular Nursing*.

[14] Tully PJ, Baker RA (2012). Depression, anxiety, and cardiac morbidity outcomes after coronary artery bypass surgery: a contemporary and practical review. *Journal of Geriatric Cardiology*.

[15] Glassman AH, Bigger JT, Gaffney M, Shapiro PA, Swenson JR (2006). Onset of major depression associated with acute coronary syndromes: relationship of onset, major depressive disorder history, and episode severity to sertraline benefit. *Archives of General Psychiatry*.

[16] Lespérance F, Frasure-Smith N, Koszycki D (2007). Effects of citalopram and interpersonal psychotherapy on depression in patients with coronary artery disease: the Canadian cardiac randomized evaluation of antidepressant and psychotherapy efficacy (CREATE) trial. *The Journal of the American Medical Association*.

[17] Huffman JC, Mastromauro CA, Sowden GL, Wittmann C, Rodman R, Januzzi JL (2011). A collaborative care depression management program for cardiac inpatients: depression characteristics and in-hospital outcomes. *Psychosomatics*.

[18] Hance M, Carney RM, Freedland KE, Skala J (1996). Depression in patients with coronary heart disease: a 12-month follow-up. *General Hospital Psychiatry*.

[19] Martens EJ, Smith ORF, Winter J, Denollet J, Pedersen SS (2008). Cardiac history, prior depression and personality predict course of depressive symptoms after myocardial infarction. *Psychological Medicine*.

[20] Kaptein KI, de Jonge P, van den Brink RHS, Korf J (2006). Course of depressive symptoms after myocardial infarction and cardiac prognosis: a latent class analysis. *Psychosomatic Medicine*.

[21] Koenig HG (2006). Depression outcome in inpatients with congestive heart failure. *Archives of Internal Medicine*.

[22] Suzuki T, Shiga T, Kuwahara K (2010). Prevalence and persistence of depression in patients with implantable cardioverter defibrillator: a 2-year longitudinal study. *Pacing and Clinical Electrophysiology*.

[23] Kroenke K, Spitzer RL, Williams JBW (2003). The patient health questionnaire-2: validity of a two-item depression screener. *Medical Care*.

[24] Kroenke K, Spitzer RL, Williams JBW (2001). The PHQ-9: validity of a brief depression severity measure. *Journal of General Internal Medicine*.

[25] Shankman SA, Nadelson J, McGowan SK, Sovari AA, Vidovich MI (2012). The predictive power of depression screening procedures for veterans with coronary artery disease. *Journal of Vascular Health and Risk Management*.

[26] Caro MA, Sowden GL, Mastromauro CA (2012). Risk factors for positive depression screens in hospitalized cardiac patients. *Journal of Cardiology*.

[27] Mallik S, Spertus JA, Reid KJ (2006). Depressive symptoms after acute myocardial infarction: evidence for highest rates in younger women. *Archives of Internal Medicine*.

[28] Strik JJMH, Lousberg R, Cheriex EC, Honig A (2004). One year cumulative incidence of depression following myocardial infarction and impact on cardiac outcome. *Journal of Psychosomatic Research*.

[29] Shanmugasegaram S, Russell KL, Kovacs AH, Stewart DE, Grace SL (2012). Gender and sex differences in prevalence of major depression in coronary artery disease patients: a meta-analysis. *Maturitas*.

[30] Busch AM, Borrelli B, Leventhal AM (2012). The relationship between smoking and depression post-acute coronary syndrome. *Current Cardiovascular Risk Reports*.

[31] Spijkerman TA, van den Brink RHS, Jansen JHC, Crijns HJGM, Ormel J (2005). Who is at risk of post-MI depressive symptoms?. *Journal of Psychosomatic Research*.

[32] Frazier L, Yu E, Sanner J (2012). Gender differences in self-reported symptoms of depression among patients with acute coronary syndrome. *Nursing Research and Practice*.

[33] Pena FM, Modenesi Rde F, Piraciaba MC (2011). Prevalence and variables predictive of depressive symptoms in patients hospitalized for heart failure. *Cardiology Journal*.

[34] Sin MK (2012). Personal characteristics predictive of depressive symptoms in Hispanics with heart failure. *Issues in Mental Health Nursing*.

[35] Lossnitzer N, Herzog W, Stork S (2012). Incidence rates and predictors of major and minor depression in patients with heart failure. *International Journal of Cardiology*.

[36] Dunkel A, Kendel F, Lehmkuhl E (2009). Predictors of preoperative depressive risk in patients undergoing coronary artery bypass graft surgery. *Clinical Research in Cardiology*.

[37] McKenzie LH, Simpson J, Stewart M (2010). A systematic review of pre-operative predictors of post-operative depression and anxiety in individuals who have undergone coronary artery bypass graft surgery. *Psychology, Health and Medicine*.

[38] Freedenberg V, Thomas SA, Friedmann E (2011). Anxiety and depression in implanted cardioverter-defibrillator recipients and heart failure: a review. *Heart Failure Clinics*.

[39] Doering LV, Moser DK, Riegel B (2010). Persistent comorbid symptoms of depression and anxiety predict mortality in heart disease. *International Journal of Cardiology*.

[40] Denollet J, Strik JJ, Lousberg R, Honig A (2006). Recognizing increased risk of depressive comorbidity after myocardial infarction: looking for 4 symptoms of anxiety-depression. *Psychotherapy and Psychosomatics*.

[41] Frasure-Smith N, Lespérance F (2008). Depression and anxiety as predictors of 2-year cardiac events in patients with stable coronary artery disease. *Archives of General Psychiatry*.

[42] de Schutter A, Lavie CJ, Milani RV (2011). Relative importance of comorbid psychological symptoms in patients with depressive symptoms following phase II cardiac rehabilitation. *Postgraduate Medicine*.

[43] Bankier B, Januzzi JL, Littman AB (2004). The high prevalence of multiple psychiatric disorders in stable outpatients with coronary heart disease. *Psychosomatic Medicine*.

[44] Martens EJ, de Jonge P, Na B, Cohen BE, Lett H, Whooley MA (2010). Scared to death? Generalized anxiety disorder and cardiovascular events in patients with stable coronary heart disease: the heart and soul study. *Archives of General Psychiatry*.

[45] Parker G, Hyett M, Hadzi-Pavlovic D, Brotchie H, Walsh W (2011). GAD is good? Generalized anxiety disorder predicts a superior five-year outcome following an acute coronary syndrome. *Psychiatry Research*.

[46] Dao TK, Chu D, Springer J (2010). Clinical depression, posttraumatic stress disorder, and comorbid depression and posttraumatic stress disorder as risk factors for in-hospital mortality after coronary artery bypass grafting surgery. *Journal of Thoracic and Cardiovascular Surgery*.

[47] Ladwig KH, Baumert J, Marten-Mittag B, Kolb C, Zrenner B, Schmitt C (2008). Posttraumatic stress symptoms and predicted mortality in patients with implantable cardioverter-defibrillators: results from the prospective living with an implanted cardioverter-defibrillator study. *Archives of General Psychiatry*.

[48] von Känel R, Baumert J, Kolb C, Cho EYN, Ladwig KH (2011). Chronic posttraumatic stress and its predictors in patients living with an implantable cardioverter defibrillator. *Journal of Affective Disorders*.

[49] Sears SF, Hauf JD, Kirian K, Hazelton G, Conti JB (2011). Posttraumatic stress and the implantable cardioverter-defibrillator patient: what the electrophysiologist needs to know. *Circulation: Arrhythmia and Electrophysiology*.

[50] Phillips AC, Batty GD, Gale CR (2009). Generalized anxiety disorder, major depressive disorder, and their comorbidity as predictors of all-cause and cardiovascular mortality: the Vietnam experience study. *Psychosomatic Medicine*.

[51] Roest AM, Zuidersma M, de Jonge P (2012). Myocardial infarction and generalised anxiety disorder: 10-year follow-up. *The British Journal of Psychiatry*.

[52] Kroenke K, Spitzer RL, Williams JBW, Löwe B (2009). An ultra-brief screening scale for anxiety and depression: the PHQ-4. *Psychosomatics*.

[53] Spitzer RL, Kroenke K, Williams JBW, Löwe B (2006). A brief measure for assessing generalized anxiety disorder: the GAD-7. *Archives of Internal Medicine*.

[54] Pizzi C, Manzoli L, Mancini S, Bedetti G, Fontana F, Costa GM (2010). Autonomic nervous system, inflammation and preclinical carotid atherosclerosis in depressed subjects with coronary risk factors. *Atherosclerosis*.

[55] Ford DE, Mead LA, Chang PP, Cooper-Patrick L, Wang NY, Klag MJ (1998). Depression is a risk factor for coronary artery disease in men: the precursors study. *Archives of Internal Medicine*.

[56] Wulsin LR, Singal BM (2003). Do depressive symptoms increase the risk for the onset of coronary disease? A systematic quantitative review. *Psychosomatic Medicine*.

[57] Carney RM, Rich MW, Freedland KE (1988). Major depressive disorder predicts cardiac events in patients with coronary artery disease. *Psychosomatic Medicine*.

[58] Kop WJ, Stein PK, Tracy RP, Barzilay JI, Schulz R, Gottdiener JS (2010). Autonomic nervous system dysfunction and inflammation contribute to the increased cardiovascular mortality risk associated with depression. *Psychosomatic Medicine*.

[59] van der Kooy K, van Hout H, Marwijk H, Marten H, Stehouwer C, Beekman A (2007). Depression and the risk for cardiovascular diseases: systematic review and meta analysis. *International Journal of Geriatric Psychiatry*.

[60] van Melle JP, de Jonge P, Spijkerman TA (2004). Prognostic association of depression following myocardial infarction with mortality and cardiovascular events: a meta-analysis. *Psychosomatic Medicine*.

[61] Frasure-Smith N, Lesperance F, Talajic M (1995). Depression and 18-month prognosis after myocardial infarction. *Circulation*.

[62] Whang W, Kubzansky LD, Kawachi I (2009). Depression and risk of sudden cardiac death and coronary heart disease in women. Results from the nurses’ health study. *Journal of the American College of Cardiology*.

[63] Frasure-Smith N, Lesperance F, Talajic M (1993). Depression following myocardial infarction: impact on 6-month survival. *The Journal of the American Medical Association*.

[64] Barth J, Schumacher M, Herrmann-Lingen C (2004). Depression as a risk factor for mortality in patients with coronary heart disease: a meta-analysis. *Psychosomatic Medicine*.

[65] Myers V, Gerber Y, Benyamini Y, Goldbourt U, Drory Y (2012). Post-myocardial infarction depression: increased hospital admissions and reduced adoption of secondary prevention measures—a longitudinal study. *Journal of Psychosomatic Research*.

[66] Ziegelstein RC, Fauerbach JA, Stevens SS, Romanelli J, Richter DP, Bush DE (2000). Patients with depression are less likely to follow recommendations to reduce cardiac risk during recovery from a myocardial infarction. *Archives of Internal Medicine*.

[67] Kurdyak PA, Gnam WH, Goering P, Chong A, Alter DA (2008). The relationship between depressive symptoms, health service consumption, and prognosis after acute myocardial infarction: a prospective cohort study. *BMC Health Services Research*.

[68] Reese RL, Freedland KE, Steinmeyer BC, Rich MW, Rackley JW, Carney RM (2011). Depression and rehospitalization following acute myocardial infarction. *Circulation: Cardiovascular Quality and Outcomes*.

[69] Leung YW, Flora DB, Gravely S, Irvine J, Carney RM, Grace SL (2012). The impact of premorbid and postmorbid depression onset on mortality and cardiac morbidity among patients with coronary heart disease: meta-analysis. *Psychosomatic Medicine*.

[70] Freedland KE, Carney RM, Rich MW (2011). Effect of depression on prognosis in heart failure. *Heart Failure Clinics*.

[71] Lange HW, Herrmann-Lingen C (2007). Depressive symptoms predict recurrence of atrial fibrillation after cardioversion. *Journal of Psychosomatic Research*.

[72] Frasure-Smith N, Lespérance F, Habra M (2009). Elevated depression symptoms predict long-term cardiovascular mortality in patients with atrial fibrillation and heart failure. *Circulation*.

[73] Beresnevait M, Benetis R, Taylor GJ, Jurnien K, Kinduris Š, Barauskien V (2010). Depression predicts perioperative outcomes following coronary artery bypass graft surgery. *Scandinavian Cardiovascular Journal*.

[74] Morone NE, Weiner DK, Belnap BH (2010). The impact of pain and depression on recovery after coronary artery bypass grafting. *Psychosomatic Medicine*.

[75] Burg MM, Benedetto MC, Rosenberg R, Soufer R (2003). Presurgical depression predicts medical morbidity 6 months after coronary artery bypass graft surgery. *Psychosomatic Medicine*.

[76] Goyal TM, Idler EL, Krause TJ, Contrada RJ (2005). Quality of life following cardiac surgery: impact of the severity and course of depressive symptoms. *Psychosomatic Medicine*.

[77] Wellenius GA, Mukamal KJ, Kulshreshtha A, Asonganyi S, Mittleman MA (2008). Depressive symptoms and the risk of atherosclerotic progression among patients with coronary artery bypass grafts. *Circulation*.

[78] Burg MM, Benedetto MC, Soufer R (2003). Depressive symptoms and mortality two years after coronary artery bypass graft surgery (CABG) in men. *Psychosomatic Medicine*.

[79] Blumenthal JA, Lett HS, Babyak MA (2003). Depression as a risk factor for mortality after coronary artery bypass surgery. *The Lancet*.

[80] van den Broek KC, Tekle FB, Habibovic M, Alings M, van der Voort PH, Denollet J (2011). Emotional distress, positive affect, and mortality in patients with an implantable cardioverter defibrillator. *International Journal of Cardiology*.

[81] de Jonge P, Honig A, van Melle JP (2007). Nonresponse to treatment for depression following myocardial infarction: association with subsequent cardiac events. *The American Journal of Psychiatry*.

[82] Carney RM, Blumenthal JA, Freedland KE (2004). Depression and late mortality after myocardial infarction in the enhancing recovery in coronary heart disease (ENRICHD) study. *Psychosomatic Medicine*.

[83] Roest AM, Martens EJ, Denollet J, de Jonge P (2010). Prognostic association of anxiety post myocardial infarction with mortality and new cardiac events: a meta-analysis. *Psychosomatic Medicine*.

[84] Howland RH, Wilson MG, Kornstein SG (2008). Factors predicting reduced antidepressant response: experience with the SNRI duloxetine in patients with major depression. *Annals of Clinical Psychiatry*.

[85] Rush AJ, Wisniewski SR, Warden D (2008). Selecting among second-step antidepressant medication monotherapies: predictive value of clinical, demographic, or first-step treatment features. *Archives of General Psychiatry*.

[86] Celano CM, Mastromauro CA, Lenihan EC, Januzzi JL, Rollman BL, Huffman JC (2012). Association of baseline anxiety with depression persistence at 6 months in patients with acute cardiac illness. *Psychosomatic Medicine*.

[87] Davidson KW, Burg MM, Kronish IM (2010). Association of anhedonia with recurrent major adverse cardiac events and mortality 1 year after acute coronary syndrome. *Archives of General Psychiatry*.

[88] Doyle F, Conroy R, McGee H (2012). Differential predictive value of depressive versus anxiety symptoms in the prediction of 8-year mortality after acute coronary syndrome. *Psychosomatic Medicine*.

[89] Damen NL, Pelle AJ, Boersma E, Serruys PW, van Domburg RT, Pedersen SS (2013). Reduced positive affect (anhedonia) is independently associated with 7-year mortality in patients treated with percutaneous coronary intervention: results from the RESEARCH registry. *European Journal of Preventive Cardiology*.

[90] Coyne JC, de Voogd JN (2012). Are we witnessing the decline effect in the type D personality literature? What can be learned?. *Journal of Psychosomatic Research*.

[91] Moyer CF, Sajuthi D, Tulli H, Williams JK (1991). Synthesis of IL-1 *α* and IL-1 *β* by arterial cells in atherosclerosis. *The American Journal of Pathology*.

[92] Ross R (1999). Atherosclerosis–an inflammatory disease. *The New England Journal of Medicine*.

[93] Hasper D, Hummel M, Kleber FX, Reindl I, Volk HD (1998). Systemic inflammation in patients with heart failure. *European Heart Journal*.

[94] Kania G, Blyszczuk P, Eriksson U (2009). Mechanisms of cardiac fibrosis in inflammatory heart disease. *Trends in Cardiovascular Medicine*.

[95] Heeschen C, Hamm CW, Bruemmer J, Simoons ML (2000). Predictive value of C-reactive protein and troponin T in patients with unstable angina: a comparative analysis. *Journal of the American College of Cardiology*.

[96] Kell R, Haunstetter A, Dengler TJ, Zugck C, Kübler W, Haass M (2002). Do cytokines enable risk stratification to be improved in NYHA functional class III patients? Comparison with other potential predictors of prognosis. *European Heart Journal*.

[97] Jimenez JA, Mills PJ (2012). Neuroimmune mechanisms of depression in heart failure. *Methods in Molecular Biology*.

[98] Kop WJ, Synowski SJ, Gottlieb SS (2011). Depression in heart failure: biobehavioral mechanisms. *Heart Failure Clinics*.

[99] Howren MB, Lamkin DM, Suls J (2009). Associations of depression with c-reactive protein, IL-1, and IL-6: a meta-analysis. *Psychosomatic Medicine*.

[100] Miller AH, Maletic V, Raison CL (2009). Inflammation and its discontents: the role of cytokines in the pathophysiology of major depression. *Biological Psychiatry*.

[101] Kop WJ, Kuhl EA, Barasch E, Jenny NS, Gottlieb SS, Gottdiener JS (2010). Association between depressive symptoms and fibrosis markers: the cardiovascular health study. *Brain, Behavior, and Immunity*.

[102] Vaccarino V, Johnson BD, Sheps DS (2007). Depression, inflammation, and incident cardiovascular disease in women with suspected coronary ischemia. The national heart, lung, and blood institute-sponsored WISE study. *Journal of the American College of Cardiology*.

[103] Katafuchi T, Kondo T, Take S, Yoshimura M (2006). Brain cytokines and the 5-HT system during poly I:C-induced fatigue. *Annals of the New York Academy of Sciences*.

[104] Katafuchi T, Kondo T, Take S, Yoshimura M (2005). Enhanced expression of brain interferon-*α* and serotonin transporter in immunologically induced fatigue in rats. *European Journal of Neuroscience*.

[105] Wirleitner B, Rudzite V, Neurauter G (2003). Immune activation and degradation of tryptophan in coronary heart disease. *European Journal of Clinical Investigation*.

[106] Skop BP, Brown TM (1996). Potential vascular and bleeding complications of treatment with selective serotonin reuptake inhibitors. *Psychosomatics*.

[107] Miyata K, Shimokawa H, Higo T (2000). Sarpogrelate, a selective 5-HT(2A) serotonergic receptor antagonist, inhibits serotonin-induced coronary artery spasm in a porcine model. *Journal of Cardiovascular Pharmacology*.

[108] Verma S, Wang CH, Li SH (2002). A self-fulfilling prophecy: C-reactive protein attenuates nitric oxide production and inhibits angiogenesis. *Circulation*.

[109] Fischer D, Rossa S, Landmesser U (2005). Endothelial dysfunction in patients with chronic heart failure is independently associated with increased incidence of hospitalization, cardiac transplantation, or death. *European Heart Journal*.

[110] Katz SD, Hryniewicz K, Hriljac I (2005). Vascular endothelial dysfunction and mortality risk in patients with chronic heart failure. *Circulation*.

[111] Cooper DC, Milic MS, Tafur JR (2010). Adverse impact of mood on flow-mediated dilation. *Psychosomatic Medicine*.

[112] Tomfohr LM, Murphy MLM, Miller GE, Puterman E (2011). Multiwave associations between depressive symptoms and endothelial function in adolescent and young adult females. *Psychosomatic Medicine*.

[113] Pizzi C, Manzoli L, Mancini S, Costa GM (2008). Analysis of potential predictors of depression among coronary heart disease risk factors including heart rate variability, markers of inflammation, and endothelial function. *European Heart Journal*.

[114] Sherwood A, Hinderliter AL, Watkins LL, Waugh RA, Blumenthal JA (2005). Impaired endothelial function in coronary heart disease patients with depressive symptomatology. *Journal of the American College of Cardiology*.

[115] Pizzi C, Mancini S, Angeloni L, Fontana F, Manzoli L, Costa GM (2009). Effects of selective serotonin reuptake inhibitor therapy on endothelial function and inflammatory markers in patients with coronary heart disease. *Clinical Pharmacology and Therapeutics*.

[116] Vikenes K, Farstad M, Nordrehaug JE (1999). Serotonin is associated with coronary artery disease and cardiac events. *Circulation*.

[117] Serebruany VL, Suckow RF, Cooper TB (2005). Relationship between release of platelet/endothelial biomarkers and plasma levels of sertraline and N-desmethylsertraline in acute coronary syndrome patients receiving SSRI treatment for depression. *The American Journal of Psychiatry*.

[118] Serebruany VL, Gurbel PA, O’Connor CM (2001). Platelet inhibition by sertraline and N-desmethylsertraline: a possible missing link between depression, coronary events, and mortality benefits of selective serotonin reuptake inhibitors. *Pharmacological Research*.

[119] Schins A, Hamulyák K, Scharpé S (2004). Whole blood serotonin and platelet activation in depressed post-myocardial infarction patients. *Life Sciences*.

[120] Arora RC, Meltzer HY (1989). Increased serotonin2 (5-HT2) receptor binding as measured by 3H-lysergic acid diethylamide (3H-LSD) in the blood platelets of depressed patients. *Life Sciences*.

[121] Hrdina PD, Bakish D, Ravindran A, Chudzik J, Cavazzoni P, Lapierre YD (1997). Platelet serotonergic indices in major depression: up-regulation of 5-HT(2A) receptors unchanged by antidepressant treatment. *Psychiatry Research*.

[122] Nemeroff CB, Knight DL, Franks J, Craighead WE, Krishnan KRR (1994). Further studies on platelet serotonin transporter binding in depression. *The American Journal of Psychiatry*.

[123] Kuijpers PMJC, Hamulyak K, Strik JJMH, Wellens HJJ, Honig A (2002). *β*-thromboglobulin and platelet factor 4 levels in post-myocardial infarction patients with major depression. *Psychiatry Research*.

[124] Serebruany VL, Glassman AH, Malinin AI (2003). Enhanced platelet/endothelial activation in depressed patients with acute coronary syndromes: evidence from recent clinical trials. *Blood Coagulation and Fibrinolysis*.

[125] Gehi A, Musselman D, Otte C, Royster EB, Ali S, Whooley MA (2010). Depression and platelet activation in outpatients with stable coronary heart disease: findings from the heart and soul study. *Psychiatry Research*.

[126] Anand IS, Fisher LD, Chiang YT (2003). Changes in brain natriuretic peptide and norepinephrine over time and mortality and morbidity in the Valsartan heart failure trial (Val-HeFT). *Circulation*.

[127] Cohn JN, Levine TB, Olivari MT (1984). Plasma norepinephrine as a guide to prognosis in patients with chronic congestive heart failure. *The New England Journal of Medicine*.

[128] Gold PW, Wong ML, Goldstein DS (2005). Cardiac implications of increased arterial entry and reversible 24-h central and peripheral norepinephrine levels in melancholia. *Proceedings of the National Academy of Sciences of the United States of America*.

[129] Frenneaux MP (2004). Autonomic changes in patients with heart failure and in post-myocardial infarction patients. *Heart*.

[130] La Rovere MT, Pinna GD, Hohnloser SH (2001). Barorefiex sensitivity and heart rate variability in the identification of patients at risk for life-threatening arrhythmias: implications for clinical trials. *Circulation*.

[131] Buccelletti F, Gilardi E, Scaini E (2009). Heart rate variability and myocardial infarction: systematic literature review and metanalysis. *European Review for Medical and Pharmacological Sciences*.

[132] Schwartz PJ, Vanoli E, Stramba-Badiale M, de Ferrari GM, Billman GE, Foreman RD (1988). Autonomic mechanisms and sudden death: new insights from analysis of baroreceptor reflexes in conscious dogs with and without a myocardial infarction. *Circulation*.

[133] Kemp AH, Quintana DS, Gray MA, Felmingham KL, Brown K, Gatt JM (2010). Impact of depression and antidepressant treatment on heart rate variability: a review and meta-analysis. *Biological Psychiatry*.

[134] Glassman AH, Bigger JT, Gaffney M, van Zyl LT (2007). Heart rate variability in acute coronary syndrome patients with major depression: influence of sertraline and mood improvement. *Archives of General Psychiatry*.

[135] Dao TK, Youssef NA, Gopaldas RR (2010). Autonomic cardiovascular dysregulation as a potential mechanism underlying depression and coronary artery bypass grafting surgery outcomes. *Journal of Cardiothoracic Surgery*.

[136] Hashimoto K (2010). Brain-derived neurotrophic factor as a biomarker for mood disorders: an historical overview and future directions. *Psychiatry and Clinical Neurosciences*.

[137] Castrén E, Rantamäki T (2010). The role of BDNF and its receptors in depression and antidepressant drug action: reactivation of developmental plasticity. *Developmental Neurobiology*.

[138] Molendijk ML, Bus BA, Spinhoven P (2011). Serum levels of brain-derived neurotrophic factor in major depressive disorder: state-trait issues, clinical features and pharmacological treatment. *Molecular Psychiatry*.

[139] Ohira K, Takeuchi R, Shoji H, Miyakawa T (2013). Fluoxetine-iduced cortical adult neurogenesis. *Neuropsychopharmacology*.

[140] Kim H, Li Q, Hempstead BL, Madri JA (2004). Paracrine and autocrine functions of brain-derived neurotrophic factor (BDNF) and nerve growth factor (NGF) in brain-derived endothelial cells. *The Journal of Biological Chemistry*.

[141] Okada S, Yokoyama M, Toko H (2012). Brain-derived neurotrophic factor protects against cardiac dysfunction after myocardial infarction via a central nervous system-mediated pathway. *Arteriosclerosis, Thrombosis, and Vascular Biology*.

[142] Hashimoto K (2013). Sigma-1 receptor chaperone and brain-derived neurotrophic factor: emerging links between cardiovascular disease and depression. *Progress in Neurobiology*.

[143] Sabino V, Cottone P, Parylak SL, Steardo L, Zorrilla EP (2009). Sigma-1 receptor knockout mice display a depressive-like phenotype. *Behavioural Brain Research*.

[144] Maurice T, Su TP (2009). The pharmacology of sigma-1 receptors. *Pharmacology and Therapeutics*.

[145] Ito K, Hirooka Y, Matsukawa R, Nakano M, Sunagawa K (2012). Decreased brain sigma-1 receptor contributes to the relationship between heart failure and depression. *Cardiovascular Research*.

[146] Kunugi H, Hori H, Adachi N, Numakawa T (2010). Interface between hypothalamic-pituitary-adrenal axis and brain-derived neurotrophic factor in depression. *Psychiatry and Clinical Neurosciences*.

[147] Bauer LK, Caro MA, Beach SR (2012). Effects of depression and anxiety improvement on adherence to medication and health behaviors in recently hospitalized cardiac patients. *The American Journal of Cardiology*.

[148] Whooley MA, de Jonge P, Vittinghoff E (2008). Depressive symptoms, health behaviors, and risk of cardiovascular events in patients with coronary heart disease. *The Journal of the American Medical Association*.

[149] May HT, Sheng X, Catinella AP, Horne BD, Carlquist JF, Joy E (2010). Antilipidemic adherence post-coronary artery disease diagnosis among those with and without an ICD-9 diagnosis of depression. *Journal of Psychosomatic Research*.

[150] Casey E, Hughes JW, Waechter D, Josephson R, Rosneck J (2008). Depression predicts failure to complete phase-II cardiac rehabilitation. *Journal of Behavioral Medicine*.

[151] McGrady A, McGinnis R, Badenhop D, Bentle M, Rajput M (2009). Effects of depression and anxiety on adherence to cardiac rehabilitation. *Journal of Cardiopulmonary Rehabilitation and Prevention*.

[152] Huffman JC, Smith FA, Fricchione GL, Januzzi JL, Nadelman S, Pirl WF (2010). Depression and failure of cholesterol lowering after acute myocardial infarction. *Primary Care Companion to the Journal of Clinical Psychiatry*.

[153] Gehi AK, Ali S, Na B, Whooley MA (2007). Self-reported medication adherence and cardiovascular events in patients with stable coronary heart disease: the heart and soul study. *Archives of Internal Medicine*.

[154] Wessel TR, Arant CB, Olson MB (2004). Relationship of physical fitness versus body mass index with coronary artery disease and cardiovascular events in women. *The Journal of the American Medical Association*.

[155] Huffman JC, Smith FA, Blais MA, Beiser ME, Januzzi JL, Fricchione GL (2006). Recognition and treatment of depression and anxiety in patients with acute myocardial infarction. *The American Journal of Cardiology*.

[156] Ziegelstein RC, Kim SY, Kao D (2005). Can doctors and nurses recognize depression in patients hospitalized with an acute myocardial infarction in the absence of formal screening?. *Psychosomatic Medicine*.

[157] Lichtman JH, Bigger JT, Blumenthal JA (2008). Depression and coronary heart disease: recommendations for screening, referral, and treatment—a science advisory from the American heart association prevention committee of the council on cardiovascular nursing, council on clinical cardiology, council on epidemiology and prevention, and interdisciplinary council on quality of care and outcomes research. *Circulation*.

[158] Elderon L, Smolderen KG, Na B, Whooley MA (2011). Accuracy and prognostic value of American heart association: recommended depression screening in patients with coronary heart disease: data from the heart and soul study. *Circulation: Cardiovascular Quality and Outcomes*.

[159] Rollman BL, Belnap BH, Mazumdar S (2012). A positive 2-item patient health questionnaire depression screen among hospitalized heart failure patients is associated with elevated 12-month mortality. *Journal of Cardiac Failure*.

[160] Thombs BD, Ziegelstein RC, Whooley MA (2008). Optimizing detection of major depression among patients with coronary artery disease using the patient health questionnaire: data from the heart and soul study. *Journal of General Internal Medicine*.

[161] Sowden G, Mastromauro CA, Januzzi JL, Fricchione GL, Huffman JC (2010). Detection of depression in cardiac inpatients: feasibility and results of systematic screening. *The American Heart Journal*.

[162] Smolderen KG, Buchanan DM, Amin AA (2011). Real-world lessons from the implementation of a depression screening protocol in acute myocardial infarction patients implications for the american heart association depression screening advisory. *Circulation: Cardiovascular Quality and Outcomes*.

[163] Ziegelstein RC, Thombs BD, Coyne JC, de Jonge P (2009). Routine screening for depression in patients with coronary heart disease. Never mind. *Journal of the American College of Cardiology*.

[164] Davidson KW, Rieckmann N, Clemow L (2010). Enhanced depression care for patients with acute coronary syndrome and persistent depressive symptoms: coronary psychosocial evaluation studies randomized controlled trial. *Archives of Internal Medicine*.

[165] Huffman JC, Mastromauro CA, Sowden G, Fricchione GL, Healy BC, Januzzi JL (2011). Impact of a depression care management program for hospitalized cardiac patients. *Circulation: Cardiovascular Quality and Outcomes*.

[166] Katon WJ, Lin EHB, Korff MV (2010). Collaborative care for patients with depression and chronic illnesses. *The New England Journal of Medicine*.

[167] Rollman BL, Belnap BH, LeMenager MS (2009). Telephone-delivered collaborative care for treating post-CABG depression: a randomized controlled trial. *The Journal of the American Medical Association*.

[168] Whooley MA (2009). To screen or not to screen? Depression in patients with cardiovascular disease. *Journal of the American College of Cardiology*.

[169] Glassman AH, O’Connor CM, Califf RM (2002). Sertraline treatment of major depression in patients with acute MI or unstable angina. *The Journal of the American Medical Association*.

[170] O’Connor CM, Jiang W, Kuchibhatla M (2010). Safety and efficacy of sertraline for depression in patients with heart failure: results of the SADHART-CHF (sertraline against depression and heart disease in chronic heart failure) trial. *Journal of the American College of Cardiology*.

[171] Berkman LF, Blumenthal J, Burg M (2003). Effects of treating depression and low perceived social support on clinical events after myocardial infarction: the enhancing recovery in coronary heart disease patients (ENRICHD) randomized trial. *The Journal of the American Medical Association*.

[172] Freedland KE, Skala JA, Carney RM (2009). Treatment of depression after coronary artery bypass surgery a randomized controlled trial. *Archives of General Psychiatry*.

